# Antibacterial Effect of Eicosapentaenoic Acid against *Bacillus cereus* and *Staphylococcus aureus*: Killing Kinetics, Selection for Resistance, and Potential Cellular Target

**DOI:** 10.3390/md15110334

**Published:** 2017-11-01

**Authors:** Phuc Nguyen Thien Le, Andrew P. Desbois

**Affiliations:** 1 School of Biotechnology, International University—Vietnam National University HCMC, Block 6, Linh Trung Ward, Thu Duc District, Ho Chi Minh City 700000, Vietnam; lntphuc@hcmiu.edu.vn; 2 Institute of Aquaculture, University of Stirling, Stirling FK9 4LA, UK

**Keywords:** antibiotic resistance, antimicrobial, fish oil, free fatty acid, omega-3, wound infections

## Abstract

Polyunsaturated fatty acids, such as eicosapentaenoic acid (EPA; C20:5n-3), are attracting interest as possible new topical antibacterial agents, particularly due to their potency and perceived safety. However, relatively little is known of the underlying mechanism of antibacterial action of EPA or whether bacteria can develop resistance quickly against this or similar compounds. Therefore, the aim of this present study was to determine the mechanism of antibacterial action of EPA and investigate whether bacteria could develop reduced susceptibility to this fatty acid upon repeated exposure. Against two common Gram-positive human pathogens, *Bacillus cereus* and *Staphylococcus aureus*, EPA inhibited bacterial growth with a minimum inhibitory concentration of 64 mg/L, while minimum bactericidal concentrations were 64 mg/L and 128 mg/L for *B. cereus* and *S. aureus*, respectively. Both species were killed completely in EPA at 128 mg/L within 15 min at 37 °C, while reduced bacterial viability was associated with increased release of 260-nm-absorbing material from the bacterial cells. Taken together, these observations suggest that EPA likely kills *B. cereus* and *S. aureus* by disrupting the cell membrane, ultimately leading to cell lysis. Serial passage of the strains in the presence of sub-inhibitory concentrations of EPA did not lead to the emergence or selection of strains with reduced susceptibility to EPA during 13 passages. This present study provides data that may support the development of EPA and other fatty acids as antibacterial agents for cosmetic and pharmaceutical applications.

## 1. Introduction

The marine-derived polyunsaturated fatty acid (PUFA) eicosapentaenoic acid (EPA; C20:5 n-3) has antimicrobial properties and there is increasing interest in developing fatty acids as new antibacterial agents, especially given the rise of bacterial pathogens with resistance against existing antibiotics [1,2,3,4]. Similar to many other PUFAs, EPA exerts potent effects against Gram-positive species, including human pathogens *Bacillus cereus* and *Staphylococcus aureus* [3]. *S. aureus* causes a multitude of clinical problems from mild skin complaints, such as impetigo, to more serious soft tissue infections, osteomyelitis, and systemic bacteraemia [5]. Meanwhile, *B. cereus* is a well-known foodborne pathogen that causes infections of the gastrointestinal tract, but this bacterium is also responsible for severe infections of the eyes, lungs, cutaneous tissues, and central nervous system [6]. Importantly, both pathogens can cause serious infections of wounds and surgical sites [5,6] and new effective treatment options are highly desirable.

In clinical and cosmetic applications, free fatty acids such as EPA could be applied topically to bolster the free fatty acids present naturally on the skin and mucosal surfaces as part of innate immunity to protect against microbial infection [3,4,7,8,9,10]. In addition to antimicrobial activities, EPA exerts beneficial anti-inflammatory actions [11] and has other positive attributes that would support its development as a new topical antibacterial agent, including wound healing properties [12], potency and perceived safety [1,4,13], and a suspected lack of acquired bacterial resistance mechanisms against this and other fatty acids [14]. However, little is known of whether or not bacteria can develop resistance quickly against this compound, or the underlying mechanisms of antibacterial action of EPA [3]. Addressing these knowledge gaps may hasten the development of EPA and other fatty acids as new topical antibacterial agents [1].

The aim of the present study was to characterize the antibacterial activity of EPA against two Gram-positive pathogens, *B. cereus* and *S. aureus*, and investigate whether the bacteria could develop reduced susceptibility to this fatty acid upon repeated exposure and determine the possible mechanism of action.

## 2. Results

**Figure 1:**
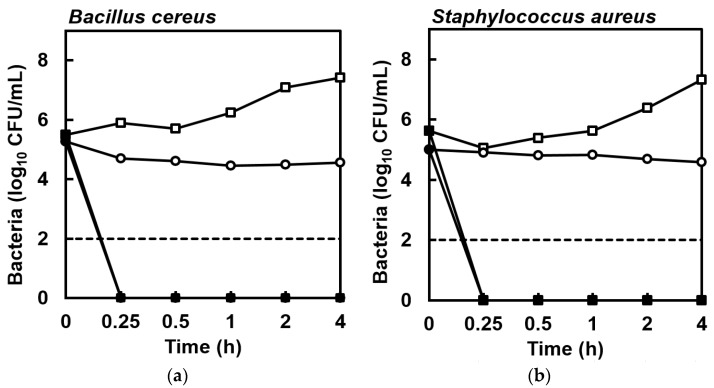
Enumeration of colonies from cell suspensions of (**a**) *Bacillus cereus* NCIMB 9373 and (**b**) *Staphylococcus aureus* Newman in MH broth (■) or PBS (●) exposed to eicosapentaenoic acid (EPA) at 128 mg/L compared to cells suspended in MH broth (□) or PBS (○) lacking EPA during 4 h at 37 °C, showing that no viable bacterial cells were detected in suspensions exposed to EPA within 15 min. The detection limit was 2 log_10_ CFU/mL (dashed line). Data are geometric mean ± standard error (not all error bars are visible); *n* = 4.

The susceptibility of *B. cereus* NCIMB 9373 and *S. aureus* Newman to EPA was assessed by broth micro-dilution according to Clinical and Laboratory Standards Institute protocols [15,16]. EPA demonstrated both growth inhibitory and bactericidal activities against *B. cereus* and *S. aureus*. The minimum inhibitory concentration (MIC) was 64 mg/L for EPA against both *B. cereus* and *S. aureus*, while the minimum bactericidal concentration (MBC) for *B. cereus* and *S. aureus* was 64 and 128 mg/L, respectively. In trials to determine kill kinetics at 128 mg/L EPA, no colonies formed by surviving cells were detected after plating 5 × 10^5^ colony forming units (CFU)/mL suspensions of *B. cereus* and *S. aureus* in Mueller-Hinton (MH) broth at 15 min or at subsequent sample times (Figure 1). As expected, some cell division occurred in the control suspensions in MH broth during the 4-h incubation (Figure 1). This experiment was repeated for cell suspensions prepared in phosphate-buffered saline (PBS) to determine whether active bacterial growth was necessary for the killing activity of EPA, but again no colonies formed by surviving cells were detected within 15 min and there was little change in CFU/mL during the 4-h incubation in the control suspensions (Figure 1).

**Figure 2:**
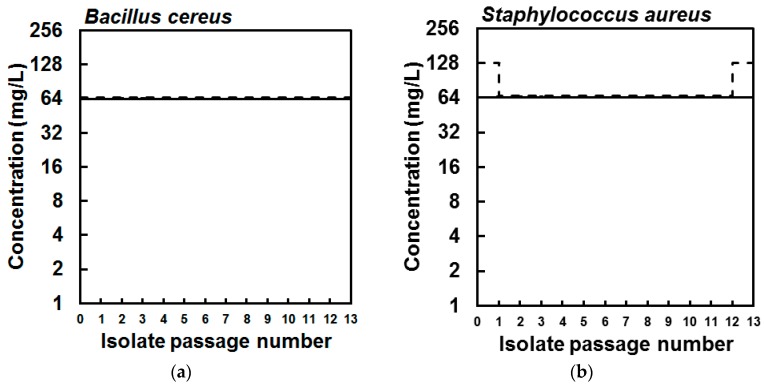
Minimum inhibitory (solid lines) and bactericidal concentration (dashed lines) values of the parent (**a**) *Bacillus cereus* NCIMB 9373 and (**b**) *Staphylococcus aureus* Newman isolates and isolates collected from each of 13 serial passages in sub-inhibitory concentrations of eicosapentaenoic acid (EPA), showing that susceptibility of the bacteria to EPA did not reduce during serial passage.

Next, to investigate the possibility to select experimentally for strains with reduced susceptibility to EPA quickly, *B. cereus* and *S. aureus* were serially passaged 13 times in the presence of sub-inhibitory concentrations of this fatty acid in the wells of a 96-well microtitre plate. At each sub-passage, the contents of the wells used to inoculate the subsequent cultures were stored at −70 °C in a cryogenic tube with 15% glycerol (*v*/*v*), so that the susceptibility of each passage isolate to EPA could be determined by MIC and compared to the parent strains. After passage of the *B. cereus* strain in sub-inhibitory concentrations of EPA, the isolates from each of 13 passages and the parent strain showed no change in susceptibility to EPA as all isolates had identical MIC and MBC values, indicating the lack of selection of *B. cereus* cells with reduced susceptibility to EPA (Figure 2). Similarly, the MIC values of the corresponding *S. aureus* passage isolates were the same as the parent strain, though the MBC value of each passaged isolate (except for the final passage isolate) was lower than the MBC of the parent strain (Figure 2). Still, there was no evidence for the selection of *S. aureus* cells with reduced susceptibility during repeated exposure to sub-inhibitory concentrations of EPA.

**Figure 3:**
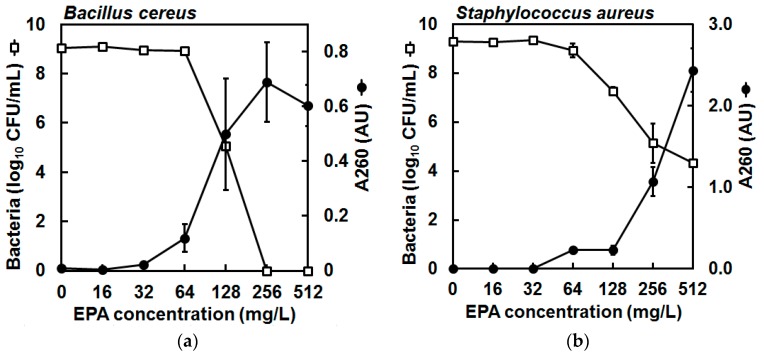
Enumeration of colonies from cell suspensions of (**a**) *Bacillus cereus* NCIMB 9373 and (**b**) *Staphylococcus aureus* Newman in PBS (□) and detection of 260-nm (A260)-absorbing material in cell-free filtrates (●) after exposure to increasing concentrations of eicosapentaenoic acid (EPA) during 30 min at 37 °C, showing that the number of viable cells in suspension reduced concomitant to the amount of A260-absorbing material in cell-free filtrates, indicating likely cell membrane perturbation and cell lysis. The bacterial inoculums at the start of incubation were 1.51 × 10^9^ ± 0.41 × 10^9^ CFU/mL (mean ± standard error) and 1.57 × 10^9^ ± 0.15 × 10^9^ CFU/mL for *B. cereus* and *S. aureus*, respectively. The carrier solvent (ethanol) had little effect on bacterial viability and at the greatest concentration of ethanol (2.56%, *v*/*v*) the bacteria recovered at 30 min was 1.31 × 10^9^ ± 0.10 × 10^9^ CFU/mL and 2.79 × 10^9^ ± 0.05 × 10^9^ for *B. cereus* and *S. aureus*, respectively (data not shown). Meanwhile, control incubations in the presence of carrier solvent (ethanol) showed that negligible quantities of A260-absorbing material were detected in cell-free filtrates (data not shown). Data are mean (geometric mean for CFU values) ± standard error (not all error bars are visible); *n* = 3 for A260 values, *n* = 2 for CFU/mL determinations (detection limit was 2 log_10_ CFU/mL); note that the secondary *y*-axis (A260) scales differ for (**a**,**b**).

Finally, to determine the mechanism of antibacterial action of EPA, leakage of 260-nm (A260)-absorbing material from the bacterial cells in suspension was quantified after exposure to increasing concentrations of EPA for 30 min, according to a protocol modified from Carson et al. [17]. The detection of A260-absorbing material can indicate membrane perturbation and an increase in membrane permeability, and these measurements were taken concomitant with bacterial viability assessments by plating of the cell suspensions on agar. The bacterial inoculums at the start of incubation were 1.51 × 10^9^ ± 0.41 × 10^9^ CFU/mL (mean ± standard error) and 1.57 × 10^9^ ± 0.15 × 10^9^ CFU/mL for *B. cereus* and *S. aureus*, respectively, and thus were considerably greater than used in the killing kinetics experiment above. The carrier solvent (ethanol) had little effect on bacterial viability and at the greatest concentration of ethanol (2.56%, *v*/*v*) the bacteria recovered at 30 min was 1.31 × 10^9^ ± 0.10 × 10^9^ CFU/mL and 2.79 × 10^9^ ± 0.05 × 10^9^ for *B. cereus* and *S. aureus*, respectively (data not shown). Control incubations in the presence of carrier solvent (ethanol) showed that negligible quantities of A260-absorbing material were detected in cell-free filtrates (data not shown). However, leakage of A260-absorbing material was detected from *B. cereus* and *S. aureus* cell suspensions that had been incubated in the presence of ≥64 mg/L EPA for 30 min, and greater concentrations of EPA led to the detection of greater quantities of A260-absorbing material released from both species of bacteria (Figure 3). Importantly, the increasing quantities of A260-absorbing material coincided with reductions in viable CFU/mL in the suspensions (Figure 3). Taken together, these observations suggest membrane disruption and probable cell lysis of the bacterial cells by EPA.

## 3. Discussion

EPA is antimicrobial and this property is being exploited in the development of new topical cosmetics and pharmaceuticals [1,2,3]; however, relatively little is known for its antibacterial mechanisms or the ease with which it is possible to select for strains with reduced susceptibility. In this present study, EPA was observed to kill rapidly two species of Gram-positive pathogen, probably by causing cell lysis, and there was little evidence for the selection of strains with reduced susceptibility after 13 passages.

In this present study, EPA inhibited the growth and killed both *B. cereus* and *S. aureus* at concentrations similar to previous reports for these and other Gram-positive species [2,3,18,19]. For *B. cereus*, MIC and MBC values were the same (64 mg/L) and killing was observed in PBS and culture medium within 15 min, indicating that actively dividing cells were not essential for bactericidal action. In conjunction with evidence of leakage of A260-absorbing material from cells at growth inhibitory and bactericidal concentrations, these data support the likely catastrophic loss of bacterial cell membrane integrity once a concentration threshold of EPA is reached. Meanwhile, for *S. aureus*, there was a two-fold difference between MIC and MBC values of EPA (64 and 128 mg/L, respectively) and EPA killed cells within 15 min, which is consistent with previous reports [2,18]. Similar to *B. cereus*, there was no evidence of the need for actively dividing cells to exert antibacterial action, as EPA killed *S. aureus* equally as effectively in PBS and MH broth. Notably, the leakage experiment showed that 2.10 × 10^4^ CFU/mL of *S. aureus* survived even at the greatest concentration of EPA (i.e., 512 mg/L) despite the release of A260-absorbing material being four times greater at this concentration than observed for *B. cereus* when no surviving cells were detected. This observation may derive from differences in the physiology of the cell membranes of these species, and it could be that EPA causes differential effects on membrane permeability of the two bacteria. Moreover, *S. aureus* may tolerate greater membrane perturbation but remain viable (for longer at least), whereas similar disruption of the *B. cereus* membrane may be lethal. Indeed, the species-specific variations in the action of EPA observed in this present study are worthy of further investigation. In addition, the composition and quantity of A260-absorbing material (typically nucleic acids [17]) in the cytoplasmic components could differ between the species and this also would need to be determined. Taken together, these observations suggest possible concentration-dependent inhibitory and bactericidal mechanisms of action [4], and EPA probably affects cell membrane-associated metabolic systems or increases permeability, ultimately leading to cell lysis. This suggestion is consistent with other studies that have proposed the cell membrane to be the main site of action for antibacterial free fatty acids, with detrimental effects caused through disruption of vital metabolic processes including cellular respiration [8,20,21,22,23] and nutrient uptake [24], or physical disturbance leading to increased permeability, leakage of cellular components [8,23,25,26,27,28], and cell lysis (reviewed by Desbois and Smith [14]).

Certain bacteria intrinsically resist the actions of fatty acids and the cell wall of Gram-positive species can confer protection against free fatty acids [25]. Some bacteria increase cell wall synthesis or decrease cell surface hydrophobicity on exposure to free fatty acids [29,30,31]. The presence of cell-membrane-stabilizing carotenoids that decrease fluidity may also reduce susceptibility to free unsaturated fatty acids [32,33]. Still, there have been few studies on the selection of bacterial strains with reduced susceptibility to antibacterial free fatty acids, particularly for Gram-positive species, and, to our knowledge, this present study is the first to perform serial passage to select for any strains with reduced susceptibility to EPA. Previously, strains of *Escherichia coli* with reduced susceptibility to caprylic acid (C8:0) or capric acid (C10:0) were selected successfully after 10 serial transfers on agar containing the fatty acids at sub-inhibitory concentrations [34], though the mechanisms underlying this phenomenon were not investigated further. Moreover, Petschow et al. [35] reported the isolation of *Helicobacter pylori* mutants on agar containing 10× MIC of the medium-chain length saturated fatty acid lauric acid (C12:0) at a rate of 10^−8^, but the susceptibility of individual colonies was not subsequently confirmed. Additionally, Obonyo et al. [36] isolated *H. pylori* cells that resisted a previously bactericidal concentration of linolenic acid (C18:3 n-3) after just three sub-cultures in a sub-bactericidal concentration of this fatty acid. In contrast, Sun et al. [37] reported no change in susceptibility to lauric acid of *H. pylori* in response to serial passage six times in sub-inhibitory concentrations. Meanwhile, Lacey and Lord [38] were unable to select stable *S. aureus* strains with reduced susceptibility to linolenic acid including mutants generated by chemical mutagenesis, and, elsewhere, no *S. aureus* strains resistant to linoleic acid (C18:2 n-6) were detected in a 5000 clone transposon insertion library [30]. The opportunity for *B. cereus* to develop resistance to EPA may be reduced by MIC and MBC values being the same as the concentration inhibiting growth is close to the concentration having a lethal effect. However, for *S. aureus*, culturable cells were detected after 30 min exposure to EPA at 512 mg/L and there exists a difference between MIC and MBC values, meaning cells are inhibited but not killed at concentrations in this window, thus potentially permitting the opportunity to select for spontaneous resistant mutants in the surviving population; nevertheless, this was not borne out in practice and serial passage of both bacteria in the presence of sub-inhibitory concentrations of EPA did not lead to the emergence or selection of strains with reduced susceptibility. These observations are consistent with the suggestion that it is more difficult to select for resistance against compounds that exert their antibacterial action by acting on multiple cellular targets and the cell membrane. This present study provides some indication of the difficulty in rapidly selecting for resistance against EPA, but future investigations will use further bacterial species and strains, undertake more passages, and employ more incremental sub-inhibitory PUFA concentrations.

To conclude, the data in this present study provide support for the development of EPA as a possible new antibacterial agent due to its favorable potency against Gram-positive pathogens, lack of rapid selection of bacterial strains with reduced susceptibility or resistance, and bactericidal mechanism of action.

## 4. Materials and Methods

### 4.1. Reagents and Bacteria

EPA (>99% purity) and culture media were purchased from Sigma-Aldrich Ltd. (Poole, Dorset, UK). An EPA stock was made in ethanol (≥99.5%) to 20 mg/mL and stored at −20 °C. All other solutions and media were made with ultrapure deionized water (Option 3; Elga, High Wycombe, Bucks, UK) and were sterilized by autoclaving at 121 °C for 15 min or by filtration (polyethersulphone, 0.22 μm; Millipore, Watford, UK). *S. aureus* Newman (gifted by Dr. Angelika Gründling, Imperial College London, UK) and *B. cereus* NCIMB 9373 were resuscitated on MH agar at 37 °C from 15% glycerol (*v*/*v*) stocks kept at −70 °C, and maintained thereafter at 4 °C.

### 4.2. Preparation of Bacterial Suspensions

Typically, bacterial suspensions were prepared from cultures that were inoculated with 3–5 colonies into 5 mL MH broth in universal bottles and incubated (37 °C, 150 rpm) until late exponential phase (determined by measuring the absorbance at 600 nm of the culture and comparing to growth curves constructed for each species; approximately 12 h). Next, bacterial cells were harvested by centrifugation (2000× *g*, 10 min, 4 °C), washed twice with PBS (for 1 L: 8 g of NaCl, 0.2 g of KCl, 1.78 g of Na_2_HPO_4_·2H_2_O, 0.24 g of KH_2_PO_4_; pH 7.4), and re-suspended to the desired CFU/mL in MH broth or PBS. The CFU/mL of the suspensions were checked by serially diluting 10 μL in PBS (in duplicate), plating on MH agar, incubating overnight (37 °C, 24 h), and performing CFU counts.

### 4.3. Assessing Antibacterial Potency

MIC values were determined by broth micro-dilution according to Clinical and Laboratory Standards Institute protocol [15]. Briefly, EPA in MH broth was serially diluted in flat-bottomed 96-well microtitre plates to final well concentrations of 4, 8, 16, 32, 64, 128, 256, and 512 mg/L (100 μL per well). Five microliters of bacterial suspension at 1 × 10^7^ CFU/mL (prepared in PBS as described in Section 4.2) was used to inoculate each well. Plates were sealed with Parafilm, incubated (37 °C, 24 h), and the lowest concentration that prevented bacterial growth visible to the naked eye was determined to be the MIC. Control wells containing either MH broth only or the volume of ethanol equal to the greatest volume in a test well were also inoculated; a non-inoculated MH broth well was also included. MBC was determined according to Clinical and Laboratory Standards Institute protocol [16] by plating 20 μL from each well showing no visible growth at 24 h on to MH agar and incubating these plates for colonies to form (37 °C, 24 h). Contents from individual wells were spread across a quarter of an agar plate and, as no attempt was made to wash away residual EPA, the effect of carryover preventing the formation of colonies cannot be dismissed. The lowest concentration of EPA that killed ≥99.9% of the initial inoculum was determined to be the MBC. MIC and MBC values were determined from duplicated series of wells.

### 4.4. Killing Kinetics

The times required for EPA at 128 mg/L to kill 5 × 10^5^ CFU/mL from exponential phase cultures of *B. cereus* and *S. aureus* were determined. Briefly, 200 μL of EPA at 128 mg/L in MH was prepared and dispensed into an Eppendorf tube, while control wells received an equal volume of carrier solvent (2.56 μL ethanol). Then, 10 μL of a cell suspension (at 1 × 10^7^ CFU/mL and prepared as described in Section 4.2) was added to each tube, before the contents were mixed by inversion and then incubated statically at 37 °C. At 15 min, 30 min, 1 h, 2 h, and 4 h, the CFU/mL in each tube was determined by serial dilution and plating of 10 μL of suspension as described in Section 4.2. Geometric means and standard errors of these values were calculated from quadruplicate trials. The experiment was repeated in PBS to determine whether active bacterial growth was necessary for the killing activity of EPA.

### 4.5. Selection of Bacterial Strains with Reduced Susceptibility to EPA

To investigate the possibility to select experimentally for strains with reduced susceptibility to EPA quickly, bacteria were serially passaged in the presence of sub-inhibitory concentrations of this fatty acid. Briefly, an MIC plate for each bacterium was prepared as described in Section 4.3, except that the final well concentrations of EPA were 16, 32, 64, and 128 mg/L. After 24–48 h incubation, 5 µL from the well showing growth at the greatest concentration of EPA was used to inoculate the wells of a new MIC plate set up such that the greatest EPA concentration was double that of the concentration in the well from which the inoculum was taken. Meanwhile, the remainder of the well contents were stored at −70 °C in a cryogenic tube with 15% glycerol (*v*/*v*). This process was continued for 13 passages for each species of bacterium. Finally, the MICs against EPA of each passage isolate and the original parent strain were determined as described in Section 4.3, once the cultures had been recovered from cryogenic stocks by culturing on MH agar (37 °C, 24 h) as described in Section 4.1.

### 4.6. Leakage of A260-Absorbing Material from Bacterial Cells

To assess bacterial membrane perturbation and increasing membrane permeability, leakage of 260-nm absorbing material was quantified from *B. cereus* and *S. aureus* cells in suspension after exposure to increasing concentrations of EPA for 30 min, according to a protocol modified from Carson et al. [17]. These measurements were performed concomitant with bacterial viability assessments by plating cell suspensions on agar. For this, EPA (solubilized in ethanol) was added to PBS and made up to 90 μL to give 16, 32, 64, 128, 256, and 512 mg/L (final volume concentrations after addition of inoculum below), while control tubes contained 90 μL PBS with ethanol concentrations corresponding to each of the respective EPA-containing tubes. Two negative control tubes contained PBS only (no EPA or ethanol). For 10 μL from each tube, A260 was determined on a NanoDrop 1000 spectrophotometer (ThermoScientific, Wilmington, DE, USA) and each of these values for the tube solutions served as the ‘blank’ when readings were to be taken again from each tube at 30 min. After this, to each tube was added 10 μL of bacterial suspension at 1.5 × 10^10^ CFU/mL, which had been prepared in PBS as described in Section 4.2, except that the cells were derived from a 500-mL shake flask culture (37 °C, 150 rpm, 24 h). Immediately, one of the negative control tubes was sampled to determine CFU/mL and the A260 of sterile-filtered supernatant. CFU/mL was determined by serial dilution and plating of 10 μL of suspension as described in Section 4.2, while the remaining 80 μL of the suspension was centrifuged (2000× *g*, 10 min). Then the supernatant was collected and passed through a 0.22-μm syringe filter (4 mm; Sterlitech, Washington, DC, USA), before the A260 of this filtrate was measured on the NanoDrop against its respective ‘blank’. Meanwhile, all other tubes were incubated (37 °C, 30 min). After incubation, the CFU/mL of unfiltered suspension and A260 of filtered suspension were determined for each tube as described above, however CFU/mL counts were performed only for the remaining negative control, each EPA-containing tube, and the tube containing the greatest volume of carrier solvent. Note that as the control incubations in the presence of carrier solvent (ethanol) showed the presence of only negligible quantities of A260-absorbing material in cell-free filtrates (data not shown), these A260 values were not subtracted from the A260 value of each respective EPA treatment reading. This experiment was repeated twice more, though CFU/mL was determined for only one of these further trials.
